# Oronasal or Intramuscular Immunization with a Thermo-Attenuated ASFV Strain Provides Full Clinical Protection against Georgia 2007/1 Challenge

**DOI:** 10.3390/v14122777

**Published:** 2022-12-13

**Authors:** Olivier Bourry, Evelyne Hutet, Mireille Le Dimna, Pierrick Lucas, Yannick Blanchard, Amélie Chastagner, Frédéric Paboeuf, Marie-Frédérique Le Potier

**Affiliations:** Agence Nationale de Sécurité Sanitaire de l’Alimentation, de l’Environnement et du Travail (Anses), Laboratoire de Ploufragan-Plouzané-Niort, BP 53, 22440 Ploufragan, France

**Keywords:** ASF, live attenuated vaccine, oronasal, intramuscular

## Abstract

African swine fever (ASF) is a contagious viral disease of suids that induces high mortality in domestic pigs and wild boars. Given the current spread of ASF, the development of a vaccine is a priority. During an attempt to inactivate the Georgia 2007/1 strain via heat treatment, we fortuitously generated an attenuated strain called ASFV-989. Compared to Georgia, the ASFV-989 strain genome has a deletion of 7458 nucleotides located in the 5′-end encoding region of MGF 505/360, which allowed for developing a DIVA PCR system. *In vitro*, in porcine alveolar macrophages, the replication kinetics of the ASFV-989 and Georgia strains were identical. *In vivo*, specific-pathogen-free (SPF) pigs inoculated with the ASFV-989 strain, either intramuscularly or oronasally, exhibited transient hyperthermia and slightly decreased growth performance. Animals immunized with the ASFV-989 strain showed viremia 100 to 1000 times lower than those inoculated with the Georgia strain and developed a rapid antibody and cell-mediated response. In ASFV-989-immunized pigs challenged 2 or 4 weeks later with the Georgia strain, no symptoms were recorded and no viremia for the challenge strain was detected. These results show that the ASFV-989 strain is a promising non-GMO vaccine candidate that is usable either intramuscularly or oronasally.

## 1. Introduction

African swine fever (ASF) is a devastating viral disease that only affects suids. ASF is asymptomatic in African wild suids, such as bush pigs or warthogs, but depending on the strain virulence, it can cause a hemorrhagic syndrome with a high lethality rate, up to 95–100%, in domestic pigs and wild boars (*Sus scrofa* sp.). Pigs infected with a highly virulent strain can display diverse symptoms such as fever, loss of appetite, depression, hemorrhages, skin lesions, vomiting and coughing. Chronic disease characterized by reduced growth and joint swelling can be seen when pigs are infected with a less virulent strain [1].

The disease is due to the African swine fever virus (ASFV), a large, enveloped, linear double-stranded DNA virus that is the only representative of the *Asfarviridae* family. Its genome of 170–194 kbp encodes for 150 to 200 proteins, of which the functions for a number of them remain unknown [2]. Based on the sequencing of C-terminus of the p72 gene, 24 genotypes of ASFV have been identified [3].

ASF was first described in Africa in 1910 [4] and remains endemic in many sub-Saharan countries. After the eradication of outbreaks due to an ASFV strain of genotype I in Europe and the Americas in the 1960–1990s, all countries outside of Africa remained free of the virus, with the exception of Sardinia, Italy, where the disease has been present since 1978 [5]. However, in 2007, a new ASFV strain of genotype II was introduced on the European continent in Georgia [6], which has further spread to the Caucasus and the Russian Federation [7], entering the European Union in 2014 [8], China in 2018 [9], and then further affecting East Asia [10]. In 2021, ASF crossed the Atlantic ocean to reach the Dominican Republic and Haiti [11], and it is still spreading among the European Union member states, as is seen with the emergence of the disease in Germany in 2021 [12] and in mainland Italy in 2022 [13].

As there is no vaccine or treatment available, the only control measures that can be applied are the culling of affected pig herds and the restriction of movement around the outbreak to contain viral spread. Regarding wild boar populations, a strategy relying on the suspension of hunting in the heart of the infected zone and any forestry activities that could disperse the animals, the division of the landscape with fences, and the drastic reduction in the number of wild boar present in the area bordering the infected zone (the so-called “white zone”), has enabled the Czech Republic and Belgium to regain their ASF-free status in two years [14]. However, this strategy could prove more difficult to apply when the affected wild boar population lives in an area that is more difficult to delimit due to its geography and landscape (mountains, wetlands, etc.), or in a region where the population undergoes multiple introductions of the virus [15,16].

Since ASF has become a global threat for pig producers, the demand for the development of a vaccine for either domestic pigs or wild boars has considerably increased over these last five years.

As recently reviewed, different vaccine development strategies have been attempted with varying success [17,18,19,20,21,22]. The administration of the inactivated virus in the absence or presence of modern adjuvants did not induce any protection, even if the candidate vaccines induced seroconversion [23,24]. Subunit vaccine-type approaches, based on recombinant proteins or plasmid DNAs, have induced little or no protection. Partial protection with a delay in clinical expression was obtained through the inoculation of ASFV DNA plasmid in the absence of seroconversion [25]. More complete protection with a marked reduction in clinical signs in 60% of the immunized pigs was obtained following immunization with a DNA library [26]. The vector-based approaches developed have also failed to protect pigs, regardless of the vector used, even if they were immunogenic [27,28], with the exception of a more recent study using a prime with a cocktail of eight recombinant adenoviruses, followed by a boost with the recombinant modified vaccinia Ankara that codes for the same eight genes [29].

Currently, it is still the live attenuated strains that induce the best protection, as already demonstrated with the naturally attenuated strains of genotype I that allow homologous or cross-protection against some other genotypes [30]. However, these attenuated strains can induce some inflammation and edema at the joint level, with negative impacts on the growth of pigs. Tentatively increasing the attenuation by introducing genetic deletion into the genomes of these naturally attenuated strains has led, depending on the case, either to a reduction in pig protection [31], or to a better protection [32], highlighting the extreme complexity of the host–virus interactions. Because of our better knowledge of the ASF virus genome, the development of virulence gene-deleted strains has emerged as a promising approach for vaccine development. As such, the deletion of multigene family (MGF) genes (MGF 505 and MGF 360) has been conducted to attenuate the Georgia strain with a good protection of immunized pigs against a Georgia virulent challenge [33]. In contrast, the deletion of other genes such as CD2v/EP402R induces inconsistent attenuation according to the parental strain [34,35]. The last gene-deleted strain developed by the USDA (ASFV-G-ΔI177L) showed very promising results, and its safety has been concluded to be satisfactory in experimental and field conditions in Vietnam [36].

Here, we report the results of experimental studies conducted on specific-pathogen-free (SPF) pigs to assess the potency and safety of the “ASFV-989”, a new live vaccine candidate generated by thermo-attenuation of the Georgia 2007/1 strain. Animals inoculated either by intramuscular or oronasal routes were fully clinically protected when challenged with the parental Georgia 2007/1 strain as soon as 2 weeks after immunization. Moreover, the Georgia genome was never detected in the blood of the immunized pigs after the challenge.

## 2. Materials and Methods

### 2.1. Viruses and Cells

The Georgia 2007/1 ASFV strain, initially isolated in 2007 from a domestic pig originating in Georgia, was kindly provided by Dr. Linda Dixon (OIE reference laboratory, The Pirbright Institute, Pirbright, UK). In order to produce an inactivated strain of ASF virus for the validation of PCR commercial kits, we heated the Georgia 2007/1 strain at 60 °C for 2 h. After the heat treatment, the viral isolation of this strain on porcine alveolar macrophages (PAMs) was unsuccessful. To further check the complete inactivation of the strain, we then inoculated two SPF pigs with this strain, which resulted in one of the pigs (pig number 989) having an attenuated form of ASF with moderate hyperthermia, low ASFV viremia, and rapid seroconversion (data not shown). The second pig inoculated with the Georgia heat-treated strain was not infected, as assessed by the absence of any ASF-related symptoms, negative PCR results, and the lack of seroconversion. The PPA/dp/FR-22/2018/180172-2 ASFV strain, hereafter named ASFV-989 (or “989”), was isolated from a blood sample collected at 7 dpi from pig 989 that was inoculated with the thermo-attenuated ASFV Georgia 2007/1 strain. Georgia 2007/1 and ASFV-989 strains were cultivated on PAMs for 3 passages and 1 passage, respectively, before being inoculated in pigs. For the characterization of the in vitro replication, PAMs were infected at a MOI of 0.1 and the cell culture supernatants were collected daily for a virus titration performed via a hemadsorption assay [37].

For the in vivo study, viruses were diluted in RPMI medium to adjust the inoculation dose to 10^3^ hemadsorbing dose 50% (HAD_50_) per pig for intramuscular (IM) inoculation and 10^4^ HAD_50_ per pig for oronasal (ON) inoculation. Immunization and challenge doses for animal experiments were confirmed by back titration.

### 2.2. Full-Genome Sequencing and Comparison

DNA was extracted from the ASFV-989 strain with the High Pure PCR Template Preparation Kit (Roche Diagnostics, Meylan, France). For library preparation, the Ion Xpress Plus Fragment Library Kit and the Ion Xpress Barcode Adapters 1–96 Kit (Thermo Fisher Scientific, Frederick, MD, USA) were used, and barcoded DNA fragments between 250 bp and 290 bp were size-selected with magnetic beads from the Agencourt AMPure XP Kit (Beckman Coulter, Villepinte, France). All samples were sequenced with the Proton Ion Torrent technology (Thermo Fisher Scientific, Frederick, MD, USA). The raw reads were cleaned with the Trimmomatic [38] 0.36 software (ILLUMINACLIP: oligos.fasta: 2:30:5:1: true; LEADING: 3; TRAILING: 3; MAXINFO: 40:0.2; MINLEN: 36). Then, a bwa [39] (version 2.2.5) alignment was performed with cleaned reads versus NCBI reference FR682468.2. The consensus sequence was created with SAMtools [40] (version 1.8) and seqtk (version 1.2) (https://github.com/lh3/seqtk accessed on 17 July 2016). The obtained sequence was annotated with Prokka (Galaxy version 1.13), and then compared to the Georgia 2007/1 strain sequence [NC_044959.1] using Seaview (version 5.0) and the seg.sites {ape} function in R 3.6.1.

Data availability: all sequence data were uploaded to the NCBI under the study accession number PRJNA784367.

### 2.3. Design of Specific PCR Systems to Detect the ASFV-989 and Georgia Strains

**Figure 1:**
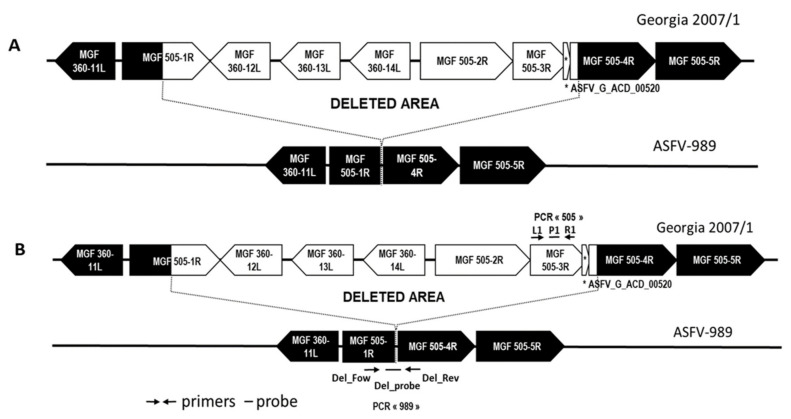
Representation of the genes and the DIVA PCR system in the deleted region of ASFV-989 strain. (**A**) Comparison of the Georgia 2007/1 and ASFV-989 genomes at the site of the deletion. (**B**) Position of the primers and probes for PCR 505 (L1, P1, and R1) to detect the Georgia 2007/1 strain and PCR 989 (Del_Fow, Del_Probe, and Del_Rev) to detect ASFV-989.

In order to have a PCR tool that is able to specifically detect the ASFV-989 or the Georgia strains in biological samples, we designed a PCR system for each of these strains. PCR 505 targets the MGF 505-3R gene of the Georgia strain with primers 505_3R_L1 TGGCAAGATCATGGTTCCCT and 505_3R_R1 ATCTGCCTCCCATGACAACA, and probe 505_3R_P1 FAM-CCCTTCCGATGCTGCTACTTTGAGTGC-TAM. PCR 989 targets the chimeric gene of the 989 strain that results from the fusion of the proximal part of MGF 505-1R and the distal part of MGF 505-4R (Figure 1B). The primers and probe sequences for PCR 989 are as follows: Del_Fow TGCTTTCAAGCCTACAACTCC, Del_Rev ATTCTCAGGGCCTCATTGGT, and Del_Probe FAM-AGCGATCCTTTGGCTGCCACC-BHQ1. First, we confirmed the specificity of each PCR system, i.e., that PCR 505 detects the Georgia strain, but not the ASFV-989 strain, and that PCR 989 detects the ASFV-989 strain, but not the Georgia strain. Then, we determined the efficacy using 10-fold dilutions of the ASFV-989 or Georgia strains. Efficacy was higher than 90% with a very good linearity (R^2^ higher than 0.99 for five dilutions) for both PCR systems.

### 2.4. In Vivo Study

In total, 65 6-week-old SPF Large White pigs were used in the three trials described in Table 1. In trial #1 and trial #2, the groups of pigs were inoculated with the ASFV-989 or Georgia strains at D0, either by the IM or ON route. Among the groups inoculated with the ASFV-989 strain (immunized), two were left unchallenged (989 ON and 989 IM long term (LT)), whereas the other groups were challenged with the Georgia strain 28 days after the ASFV-989 inoculation. Control pigs (nonimmunized and nonchallenged) were also included in each trial. In trial #3, in order to evaluate the onset of protection induced by the ASFV-989 inoculation, pigs were challenged with the Georgia strain 14 days after immunization, with the challenge carried out through the same route as for the ASFV-989 inoculation (IM or ON).

Pigs were identified individually and each group of pigs were housed in separated rooms in the air-filtered biosafety level 3 animal facilities at Anses-Ploufragan. All pigs were monitored daily for rectal temperature and clinical signs of ASFV infection, and they were weighed once per week as previously described [30]. Blood samples were collected in EDTA tubes and dry tubes before inoculation or challenge, then twice a week during the first 2 weeks after inoculation/challenge, and finally, once a week during the remaining period of the follow-up for ASFV genome and antibody detection. Blood samples were also collected on heparin tubes at D13 and D27 after ASFV-989 inoculation to monitor the cell mediated immune response. At the end of the experiment, or at earlier stages for animal welfare reasons, pigs were euthanized by anesthetic overdose and then exsanguinated.

Ethics statement: animal experiments were authorized by the French Ministry for Research (project N° 2019030418445731) and approved by the national ethics committee (authorization N° 19-018#19585).

### 2.5. Virological and Immunological Assays

Real-time PCR: The assessment of the ASFV viremia during the first week after inoculation was performed with a pan-ASFV real-time PCR, as previously described [41], using DNA extracted from 100 μL of EDTA blood samples with the NucleoSpin^®^ 8 Virus (Macherey-Nagel, Düren, Germany). The ASFV genomic load was determined by means of a standard viral range (with a known HAD_50_ titer) diluted in negative blood. The results were expressed as equivalent (eq) HAD_50_/mL of blood. The long term follow-up of the ASFV-989 viremia and the specific detection of the ASFV-989 or Georgia genome in immunized and then challenged pigs were performed with a DIVA PCR system (PCR 989 and PCR 505) developed for this study and described above. The ASFV genomic load was determined using a standard viral range of strain ASFV-989 for PCR 989 and of strain Georgia 2007/1 for PCR 505.

ELISA: antibodies to ASFV p32 were measured in serum using a commercial competition ELISA kit according the manufacturer’s instructions (ID Screen ASFV Competition, IDVET, Montpellier, France).

ELISPOT IFNγ: ASFV-specific IFNγ-secreting cells (IFNγ-SCs) were quantified, as previously described [30], using a 16 h ASFV stimulation of 4 × 10^5^ PBMCs with a multiplicity of infection of 0.2 for the Georgia strain. The number of spots per well was counted using an ImmunoSpot S6 UV Analyzer (CTL, Shaker Heights, OH, USA).

## 3. Results

### 3.1. Genome Sequence and In Vitro Characterization of ASFV-989

#### 3.1.1. Full-Genome Sequencing of the ASFV-989 Strain

The ASFV-989 strain was isolated from a blood sample collected from pig #989 7 days after inoculation with the thermo-attenuated ASFV Georgia strain. The ASFV-989 strain was further amplified once on PAMs and submitted to full-genome sequencing.

A comparison of the ASFV-989 and Georgia 2007/1 (NC_044959.1) full-genome sequences revealed two nucleotide differences out of all ORFs, including one substitution in MGF 505-11L generating an amino acid change in position 314 (Met → Thr) and a major deletion of 7458 nucleotides in the ASFV-989 strain (between nucleotide positions 29,439 and 36898) that corresponds to the partial deletion of MGF 505-1R and 505-4R and the complete deletion of MGF 360-12L, 360-13L, 360-14L, 505-2R, 505-3R, and ASFV_G_ACD_00520 (Figure 1A).

Use of PCR 989 (Figure 1B) on the initial Georgia 2007/1 viral suspension prior to heat treatment gave negative results, demonstrating the absence of the ASFV-989 genome in the Georgia 2007/1 strain stock (data not shown).

#### 3.1.2. In vitro Replication of the ASFV-989 and Georgia Strains

**Figure 2:**
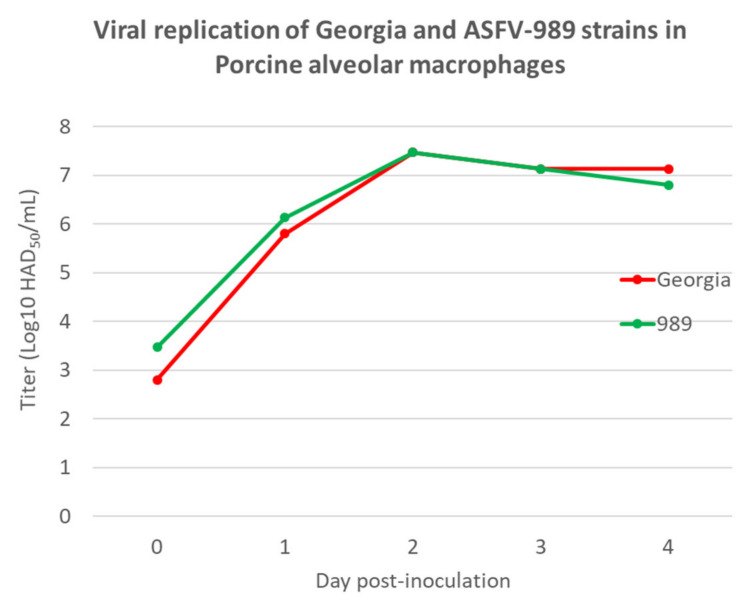
Viral replication of Georgia 2007/1 and ASFV-989 strains in porcine alveolar macrophages (titer in Log10 HAD_50_/mL, time in days post-inoculation).

To characterize the in vitro replication of the ASFV-989 strain in comparison to its parental strain Georgia, porcine alveolar macrophages (PAMs) were infected with Georgia or ASFV-989 strains and the replication kinetics were followed over 4 days. As depicted in Figure 2, the replication kinetics were similar for the ASFV-989 and the Georgia strains, with an increase in the viral titer from D0 to D2 post-inoculation, followed by a plateau from D2 to D4.

### 3.2. In Vivo Characterization of the Virulence of ASFV-989 Strain in Comparison to Georgia Strain

To characterize the virulence level of the ASFV-989 strain, we conducted two initial in vivo trials, during which we inoculated pigs either with the ASFV-989 or Georgia strains by the intramuscular (IM) or oronasal (ON) route (Table 1).

In the first step, we compared the clinical and virological parameters for pigs inoculated through the ON or IM route with the ASFV-989 strain to pigs receiving the virulent Georgia strain by the same routes. To increase the number of pigs per condition, the data with the same experimental conditions from trial #1 and trial #2 were aggregated. The experimental groups corresponding to each condition are mentioned in each figure legend.

#### 3.2.1. Clinical and Zootechnical Data

- Intramuscular inoculation

**Figure 3:**
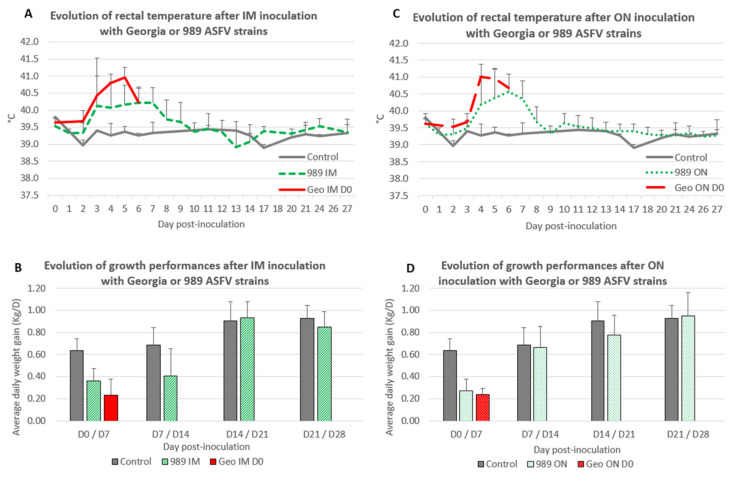
Evolution of rectal temperature and growth performances after Georgia or ASFV-989 strain inoculation. (**A**) Evolution of rectal temperature (°C) after IM inoculation with Georgia or ASFV-989 strains (time in days post-inoculation). Control—data from group A1; 989 IM—data from groups C and D; Geo IM—data from group E that were euthanized/died at 6 dpi. (**B**) Evolution of growth performances: average daily weight gain (Kg/D) after IM inoculation with Georgia or ASFV-989 strains (time in days post-inoculation). Control—data from groups A1 and A2; 989 IM—data from groups C and D; Geo IM—data from group E. (**C**) Evolution of rectal temperature (°C) after ON inoculation with Georgia or ASFV-989 strains (time in days post-inoculation). Control—data from group A1; 989 ON—data from groups B, H, and I; Geo ON—data from group F that were euthanized/died at 6 dpi. (**D**) Evolution of growth performances: average daily weight gain (Kg/D) after ON inoculation with Georgia or ASFV-989 strains (time in days post-inoculation). Control—data from group A1 and A2; 989 ON—data from groups B, H, and I; Geo ON—data from group F.

After IM inoculation with the Georgia strain, the infected pigs (Geo IM D0: group E) displayed the first hyperthermia at D3 post-inoculation (PI); then, they showed a peak of hyperthermia 2 days later (41.0 ± 0.3 °C at D5 PI, Figure 3A) and developed the typical acute ASF-related symptoms. All of them had to be euthanatized at D6 PI.

The pigs inoculated intramuscularly with the ASFV-989 strain (989 IM: groups C and D) also showed the first hyperthermia at D3 PI, but developed lower hyperthermia levels (40.2 ± 0.6 °C at D5 PI, Figure 3A) and limited symptoms.

Among the 11 pigs inoculated IM with the ASFV-989 strain, only 2 animals developed more severe symptoms (weight loss, breathing difficulties, and joint swelling), and had to be euthanatized at D9 and D13 PI. An increase in rectal temperature in the IM ASFV-989 inoculated pigs was accompanied with a decrease in growth performance during the 2 weeks after inoculation (Figure 3B). The average daily weight gain (ADWG) between D0 and D7 PI was 0.36 ± 0.12 kg/D for 989 IM versus 0.64 ± 0.10 kg/D for the control, and the ADWG between D7 and D14 PI was 0.41 ± 0.24 kg/D for 989 IM versus 0.69 ± 0.16 kg/D for the control.

- Oronasal inoculation

Following ON inoculation of the Georgia strain, the pigs (Geo ON D0: group F) displayed the same severe symptoms as those IM inoculated, showing a peak of hyperthermia (41.0 ± 0.4 °C) at D4 PI (Figure 3C). All the pigs also had to be euthanatized at D6 PI. On the other hand, pigs inoculated through the ON route with the ASFV-989 strain (989 ON: groups B, H, and I) developed a lower fever (40.6 ± 0.5 °C at D6 PI) (Figure 3C) and limited symptoms, and all the pigs survived the infection. ON-inoculated Pigs with the ASFV-989 strain also displayed a drop in growth performance (ADWG between D0 and D7 PI: 0.27 ± 0.11 kg/D for 989 ON versus 0.64 ± 0.10 kg/D for control), but only during the first week after inoculation (Figure 3D).

#### 3.2.2. Virological Data

**Figure 4:**
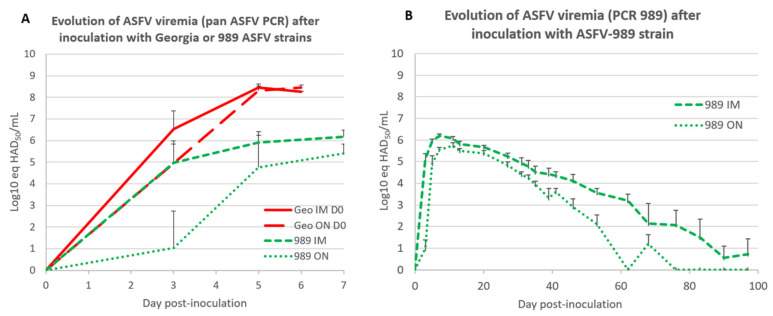
Evolution of ASFV viremia after Georgia or ASFV-989 strain inoculation. (**A**) Evolution of ASFV viremia (Log10 eq HAD_50_/mL) after inoculation with Georgia or ASFV-989 strains using a qPCR test [41] that detect both ASFV-989 and Georgia strains (time in days post-inoculation). Geo IM—data from groups E and F; Geo ON—data from groups F and J; 989 IM—data from groups C and D; 989 ON—data from groups B, H, and I. (**B**) Evolution of ASFV viremia (PCR 989, Log10 eq HAD_50_/mL) after IM or ON inoculation with ASFV-989 strain (time in days post-inoculation). 989 IM—data from groups C and D; 989 ON—data from groups B, H, and I.

To further characterize the attenuated nature of the ASFV-989 strain, we then evaluated the viremia level for the ASFV-989 strain in comparison to the Georgia strain over the first week of infection for pigs inoculated by the IM or ON route, using a PCR test that detect both strains [41]. As depicted in Figure 4A, the viremia level for the ASFV-989 strain is markedly lower than for the Georgia strain, with a 100-fold lower viral load for the ASFV-989 strain. For the IM-inoculated pigs that received the Georgia strain, the ASFV viremia at D5 PI was 8.5 ± 0.2 log10 eq HAD_50_/mL, but only 5.9 ± 0.3 log10 eq HAD_50_/mL for the animals inoculated via the same route with the ASFV-989 strain. Interestingly, the pigs inoculated through the ON route with the ASFV-989 strain displayed a lower viremia than those IM inoculated (at D5 PI, 4.7 ± 1.6 log10 eq HAD_50_/mL for ON versus 5.9 ± 0.3 log10 eq HAD_50_/mL for IM inoculation route).

In order to more precisely describe the ASFV viremia profile in pigs inoculated with the ASFV-989 strain (either IM or ON), we followed the viremia for the ASFV-989 strain for up to 100 days with the PCR 989 in the animals of groups B and C. The data shown in Figure 4B confirmed the lower level of viremia for the ON inoculation route (at D7 PI, 6.2 ± 0.3 log10 eq HAD_50_/mL for IM versus 5.5 ± 0.4 log10 eq HAD_50_/mL for ON), with an earlier extinction of viremia for the ON inoculation route (at D76 PI: 0/4 pigs viremic for ON versus 3/4 still viremic for IM).

### 3.3. Development of ASFV-Specific Immune Response after Inoculation with Georgia or ASFV-989 Strains

**Figure 5:**
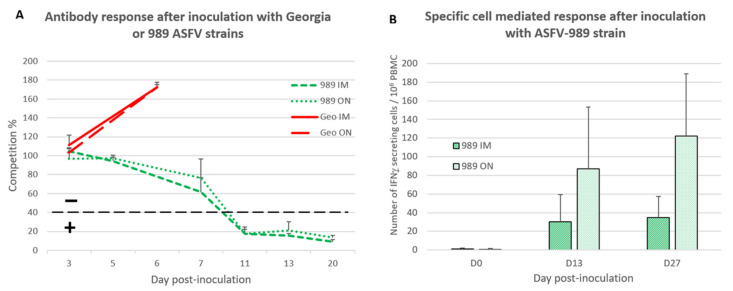
Development of the ASFV-specific immune response after ASFV-989 or Georgia strain inoculation. (**A**) Detection of ASFV-specific antibodies (competition ELISA) after Georgia or ASFV-989 strain inoculation (time in days post-inoculation). The dotted line indicates the positivity threshold of ELISA test. “–“ indicates seronegative samples, “+” indicates seropositive ones. 989 IM—group C; 989 ON—group B; Geo IM—group E; Geo ON—group F. (**B**) Detection of the cell-mediated immune response (number of IFNγ-secreting cells/10^6^ PBMC) after inoculation with ASFV-989 strain (time in days post-inoculation). 989 IM—data from groups C and D; 989 ON—data from groups B, H, and I.

After investigating the clinical and virological parameters, we explored the specific immune response that might arise after ASFV-989 inoculation. As expected, the rapid fatal evolution of the Georgia strain infection precluded the development of an ASFV-specific antibody response no matter the inoculation route (Figure 5A), with a competition percentage staying high above the positive threshold of 40%. In contrast, the pigs inoculated with the ASFV-989 strain developed an antibody response with seroconversion occurring between D7 and D11 PI for both IM and ON inoculation routes (Figure 5A).

The cell-mediated immune (CMI) response specific to ASFV was evaluated by using the ELISPOT IFNγ among IM- or ON-inoculated pigs that received the ASFV-989 strain. A CMI response specific to the ASFV virus was detected as soon as D13 PI and confirmed at D27 PI (Figure 5B). The level of the CMI response was stronger for the ON group (122 ± 67 IFNγ-SCs/10^6^ PBMC at D27 PI) compared to the IM one (35 ± 23 IFNγ-SCs/10^6^ PBMC at D27 PI).

### 3.4. Protective Effect of ASFV-989 Strain Inoculation against a Challenge with the Georgia Strain Occurring 4 Weeks Later

Considering that the pigs inoculated with the ASFV-989 strain developed a specific immune response, we then evaluated if the immunized pigs may be protected against an ASFV challenge with the virulent Georgia strain 1 month after immunization (Table 1).

#### 3.4.1. Clinical and Zootechnical Data

**Figure 6:**
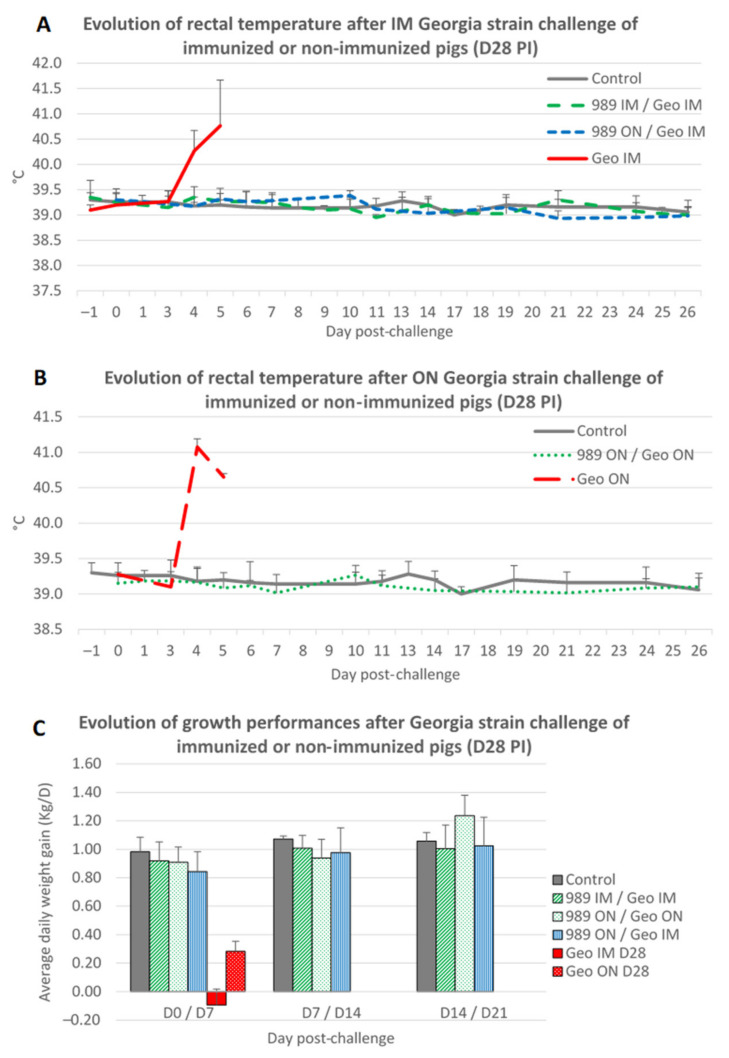
Evolution of rectal temperature and growth performance after Georgia challenge occurring 28 days after immunization. **(A**) Evolution of rectal temperature (°C) after IM challenge with Georgia ASFV strain (time in days post-challenge). Control—data from group A2; 989 IM/Geo IM—data from group D; 989 ON/Geo IM—data from group I; Geo IM—data from group G. (**B**) Evolution of rectal temperature (°C) after ON challenge with Georgia ASFV strain (time in days post-challenge). Control—data from group A2; 989 ON/Geo ON—data from group H; Geo ON—data from group J. (**C**) Evolution of growth performance: average daily weight gain (Kg/D) after IM or ON challenge with Georgia ASFV strain (time in days post-challenge). Control—data from group A1 and A2; 989 IM/Geo IM—data from group D; 989 ON/Geo ON—data from group H; 989 ON/Geo IM—data from group I; Geo ON—data from group J.

As shown previously, a challenge with the Georgia strain delivered through the ON or IM route induced rapid hyperthermia and severe symptoms in nonimmunized pigs (Figure 6A,B), leading to the compassionate euthanization of the pigs (groups G and J). On the contrary, none of the pigs immunized with the ASFV-989 strain and challenged with the Georgia strain developed any hyperthermia or ASF-related symptoms, and all survived the Georgia challenge (groups D, H, and I).

In terms of growth performance, the Georgia challenge had no impact on the ASFV-989-immunized pigs in contrast to nonimmunized animals that demonstrated a quick drop in their average daily weight gain (Figure 6C).

#### 3.4.2. Virological Data

Having determined that the pigs immunized with the ASFV-989 strain were fully clinically protected against the Georgia challenge, we then assessed the effect of ASFV-989 immunization at the virological level. Using both the PCR 505 (to detect the Georgia strain) and the PCR 989 (to detect the ASFV-989 strain), we evaluated the Georgia and ASFV-989 viremia in pigs that were previously immunized or not with ASFV-989, and then were challenged with the Georgia strain.

As shown in Table 2 and Table 3, both groups of nonimmunized pigs displayed a quick increase in Georgia viremia after the challenge (at D5 PC: 8.9 ± 0.2 log10 eq HAD_50_/mL for Georgia IM and 9.0 ± 0.4 log10 eq HAD_50_/mL for Georgia ON). On the contrary, the Georgia genome was not detected in the blood of any of the ASFV-989-immunized pigs, whatever the route of immunization or the route of challenge. As shown previously in Figure 4B, the PCR 989 results (Table 2 and Table 3) confirmed that the ASFV-989 viremia could be detected for at least 6 weeks after immunization.

In tissue samples collected at necropsy (40 days post-challenge), the Georgia genome was not detected in any of the pigs immunized with the ASFV-989 strain through the IM route (Table 4). Among the pigs immunized with the ASFV-989 strain through the ON route, the Georgia genome was detected in the tissues (tonsil, spleen, or hepato-gastric lymph node) of 4 out of 12 pigs (2 in group H and 2 in group I). The genome of the ASFV-989 strain was detected in 11/16 pigs, and most of the time, with only one positive tissue and high Ct values (Table 4).

### 3.5. Protective Effect of ASFV-989 Strain Inoculation against a Challenge with the Georgia Strain Occurring 2 Weeks Later

Having demonstrated that ASFV-989 inoculation was able to induce a strong protection against a Georgia challenge that occurred 4 weeks later, we then questioned if the same protection could be acquired as soon as 2 weeks after immunization. Thus, two groups of pigs were inoculated with the ASFV-989 strain by the IM or ON route, and then challenged with the Georgia strain 2 weeks later via the same route (Table 1, groups K and L).

**Figure 7:**
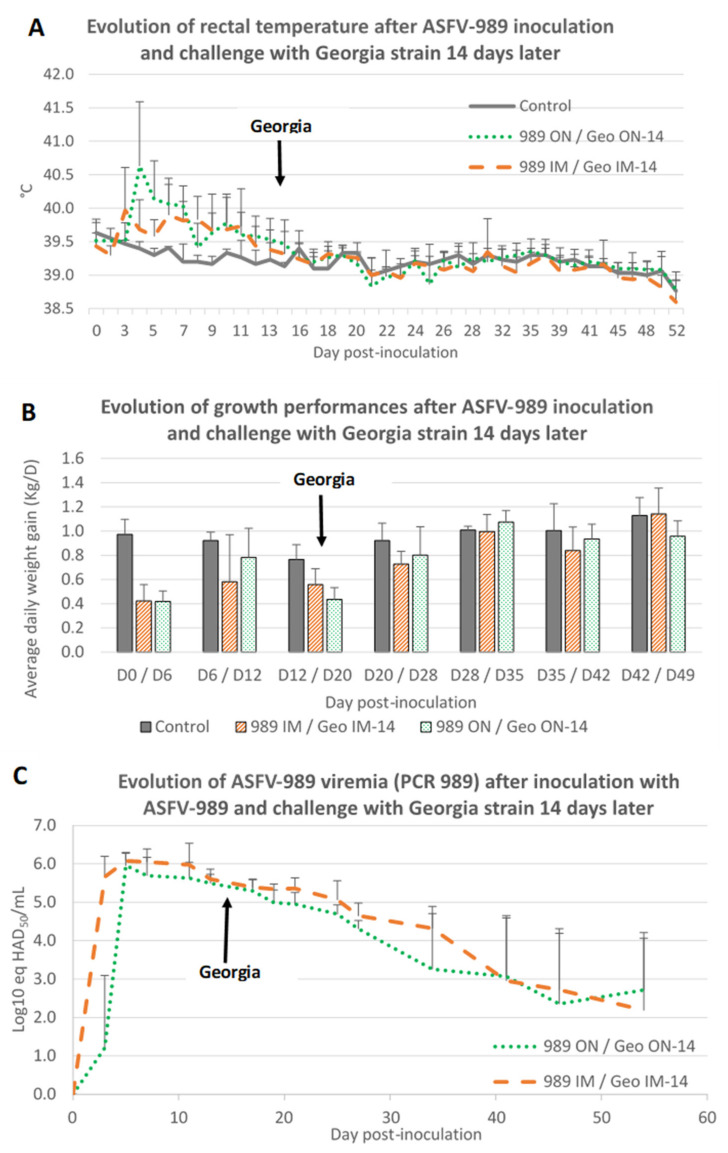
Evolution of rectal temperature, growth performance, and ASFV viremia after ASFV-989 inoculation and Georgia challenge occurring 14 days after inoculation. (**A**) Evolution of rectal temperature (°C) (time in days post-inoculation). (**B**) Evolution of growth performance: average daily weight gain (Kg/D) (time in days post-inoculation). (**C**) Evolution of ASFV viremia (PCR 989, Log10 eq HAD_50_/mL) (time in days post-inoculation). Control—data from group A3; 989 IM/Geo IM-14—data from group K; 989 ON/Geo ON-14—data from group L.

As seen in trial #1 and #2, inoculation of pigs with the ASFV-989 strain induced a period of hyperthermia beginning at day 3 post-inoculation and lasting around 1 week (Figure 7A). This hyperthermia was accompanied by a decrease of growth performances during 1 to 2 weeks (Figure 7B). Among the pigs inoculated intramuscularly with the ASFV-989 strain, one animal developed a subacute form of ASF (weight loss, tremor and ataxia) and died at D12 post-inoculation.

Following the Georgia challenge (at 14 days post-immunization) no symptoms, no hyperthermia as well as no impact on growth performances were noticed for any of the ASFV-989 immunized pigs (Figure 7A,B).

At the virological point of view, following ASFV-989 inoculation, all the pigs displayed a detectable viremia for the attenuated strain which was detected faster and with a slightly higher level for IM inoculated pigs as compared to ON inoculated ones (Figure 7C). After the Georgia challenge, no genome of the Georgia strain (PCR 505) was detected in the blood (Table 5) or in the tissues (Table 6) of the immunized pigs.

### 3.6. Global Safety and Efficacy of ASFV-989 as a Candidate Vaccine Strain

**Figure 8:**
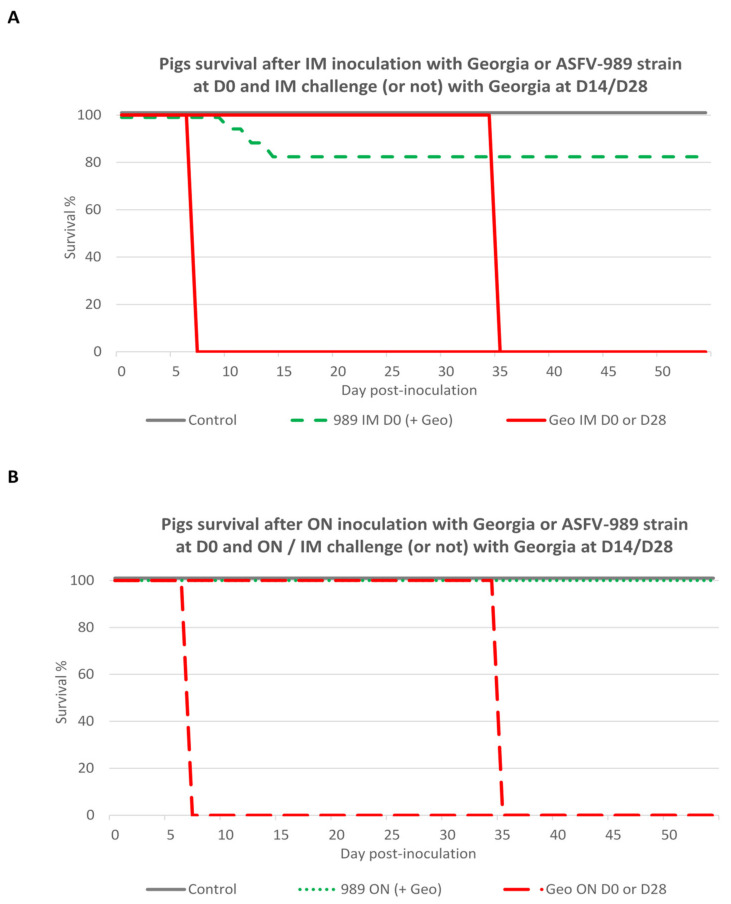
Survival curves for pigs inoculated with Georgia or ASFV-989 strains at D0 and then challenged or not with the Georgia strain. (**A**) Survival curves for pigs inoculated with ASFV-989 or Georgia by intramuscular route and challenged (or not) at D14 or D28 with Georgia strain via the same route (time in days post-inoculation). Control—data from group A1, A2, and A3; 989 IM D0—data from groups C, D, and K; Geo IM D0 or D28—data from groups E and G. (**B**) Survival curves for pigs inoculated with ASFV-989 or Georgia by oronasal route and challenged (or not) at D14 or D28 with Georgia strain via IM or ON route (time in days post-inoculation). Control—data from group A1, A2, and A3; 989 ON D0—data from groups B, H, I and L; Geo ON D0 or D28—data from groups F and J.

To sum up the results of our three in vivo trials, we drew survival curves for the pig groups inoculated or not with the attenuated or virulent strain, and then challenged or not with the Georgia strain (Figure 8). Among the pigs inoculated with the Georgia strain at D0, all the pigs died or had to be euthanized within one week after infection, whatever the route of inoculation. For the pigs inoculated with the ASFV-989 strain intramuscularly, 3 animals out of 17 died or had to be euthanized between D10 and D14 post-inoculation (global survival rate of 82%). In contrast, none of the pigs inoculated with the ASFV-989 strain by the oronasal route died (global survival rate of 100%). Whatever the route used (IM or ON) for the challenge, all the nonimmunized pigs challenged at D28 died (or had to be euthanized). In contrast, all the pigs immunized with the ASFV-989 strain, no matter the immunization and challenge route or the delay between immunization and challenge (14 or 28 days), survived the challenge, endorsing the full protection afforded by the ASFV-989 immunization.

## 4. Discussion

We did not expect to generate the ASFV-989 strain when we carried out the protocol of thermo-inactivation by incubating a tube of ASFV Georgia 2007/1 in a wet bath for two hours at 60 °C. Even if it was not possible to isolate the virus on PAMs after this heat treatment, one of the two inoculated SPF pigs developed moderate hyperthermia, ASFV viremia, and an antibody response. From a blood sample collected from pig 989 at 7 dpi, we could isolate a new attenuated strain that we named “ASFV-989”.

Thermo-attenuation of ASFV has never been reported before. The only other virus for which we found reports of heat treatment attenuation is the infectious bronchitis virus (IBV) [42,43]. In this case, the thermo-attenuation process consists of repeated combinations of heat treatment at 56 °C, followed by inoculation of embryonated eggs. For IBV, the mechanism underlying the thermo-attenuation would be related to a higher thermal resistance of low-virulence isolates.

In the case of the thermo-attenuation of ASFV we reported here, we speculate that the underlying mechanism may be different as the PCR 989 performed on the initial Georgia 2007/1 viral suspension submitted to heat treatment gave negative results, indicating that the ASFV-989 strain was not present before heat treatment. Even if highly speculative, we could hypothesize that the heat treatment may have induced a fragility of the viral genome that conducted to the deletion in the MGF 505/360 genes. Considering that the genetic alteration of the virus due to heat treatment is certainly random, we can assume that reproducing the procedure by applying the same heat treatment to the same ASFV isolate would probably not induce the same results.

Through full-genome sequencing, we identified in the ASFV-989 strain a deletion of 7458 nucleotides corresponding to the partial deletion of MGF 505-1R and 505-4R and the complete deletion of MGF 360-12L, 360-13L, 360-14L, 505-2R, 505-3R, and ASFV_G_ACD_00520. It is worth mentioning that the deleted region in the ASFV-989 strain is very close to the one deleted in the ASFV-G-ΔMGF attenuated strain previously described by O’Donnel et al. [33], which encompasses MGF 505-1R, 360-12L, 360-13L, 360-14L, 505-2R, and 505-3R. The nonessential genes deleted in both strains are known to be involved in ASFV immune evasion, especially in the suppression of the type I interferon response [44,45]. The kinetics of multiplication of this new strain were then compared to Georgia 2007/1 on PAMs, and we showed that the ASFV-989 strain demonstrated the same capacity to replicate in the host cells as the parental strain.

As ASF has become a global threat to pig production, a vaccine that could be applied via an intramuscular route in commercial pig farms or by an oral route to backyard or wild boar populations is of paramount interest for the pork industry as a complementary tool to control the disease. For this reason, we directly compared the two routes of inoculation, intramuscular (IM) and oronasal (ON), in our SPF pigs.

Only three out of seventeen ASFV-989 IM-inoculated pigs displayed some severe clinical signs and died or had to be euthanized. All the other pigs developed lower fever, limited symptoms, and a drop in growth performance in the two first weeks post-inoculation, and then they recovered. Even if genetically close, the virulence of the ASFV-989 strain seems slightly higher than the one of the ASFV-G-ΔMGF strain, as O’Donnel et al. did not detect any clinical signs after IM inoculation of 10^4^ HAD_50_ of the ASFV-G-ΔMGF strain [33]. This difference in virulence level could be due to the slight genetic differences between the two deleted strains. Indeed, as highlighted by Rathakrishnan and al. [45], the differential deletion of only a few MGF genes can significantly modify the virulence level of ASFV strains.

Another explanation for the relatively high level of residual virulence measured for the ASFV-989 strain could be linked to our SPF pig infection model. Our SPF pigs were previously shown to be highly sensitive to ASFV when they were inoculated with attenuated strains such as OURT88/3 [30] or E75CV1 [26], possibly in relation to their relatively poor microbiota diversity compared to conventional farm pigs [46].

Pigs inoculated oronasally with the ASFV-989 strain displayed a drop in growth performance during the first week post-inoculation, and then recovered and remained clinically normal until the end of the experiment, with no pig mortality in this group. IM- and ON-inoculated pigs showed a peak of ASFV-989 viremia at 7 days post-inoculation, but this peak was lower in ON-inoculated compared to IM-inoculated pigs. The duration of the viremia was also shorter for ON-inoculated than for IM-inoculated pigs. Here, a link might be made between the lower virulence of the ASFV-989 strain in the pigs inoculated via the ON route and the lower ASFV viremia measured in these animals. Interestingly, we also measured a higher level of a cell-mediated immune response in pigs inoculated through the ON route, which could explain the better control of the ASFV-989 viremia in these animals.

After the challenge with the Georgia 2007/1 strain at 4 weeks post-immunization, all the pigs remained clinically healthy no matter the inoculation route. No Georgia 2007/1 viremia was detected in any pigs, even among the pigs immunized through the ON route that were further challenged intramuscularly, which suggests that the strong vaccine efficacy is unrelated to the immunization or the challenge route. This high level of clinical and virological protection against the Georgia 2007/1 challenge was also reached when reducing the delay between the immunization and challenge to 2 weeks, suggesting a rapid onset of immunity that could be linked to the observed rapid seroconversion, but also to the induction of an ASFV-specific, cell-mediated immune response in less than 14 days post-immunization. Indeed, as previously described by Takamatsu et al. [47], cellular immune responses are probably involved in the strong clinical and virological protection induced by the ASFV-989 immunization.

In terms of vaccine efficacy, the ASFV-989 strain seems to show a higher efficiency compared to the ASFV-G-ΔMGF strain since the Georgia strain was detected after the challenge in 30 to 40% of the pigs immunized with the ASFV-G-ΔMGF strain [33]. Regarding the safety/efficacy balance, we can consider the ASFV-G-ΔMGF strain to have very good safety but imperfect efficacy, whereas the ASFV-989 strain has perfectible safety but very strong efficacy. As mentioned previously, these apparent differences between the phenotype of the two strains could be also due to differences between the pig models used to assess each strain.

The next step will be to adapt the ASFV-989 strain to grow on a permanent cell line, as it is difficult to scale-up to the industrial production a strain cultivated on primary cells such as macrophages. However, the adaptation of the strain to a cell line can further attenuate it and modify its efficacy; thus, other in vivo experiments will be necessary to check if the adaptation has led to better attenuation while keeping its efficacy, as performed by Borca et al. for the ASFV-G-ΔI177L vaccine candidate [48].

Based on the results presented in this report, the ASFV-989 strain can be considered as a good vaccine candidate because of its high efficacy in inducing protection, even by the oronasal route, opening up a way for its potential usage through oral vaccination in wild-boar-affected populations, as carried out successfully in the past in Western Europe to eradicate classic swine fever [49]. Moreover, the differential PCR that we have developed would allow for differentiating between vaccinated and infected animals, which is an important step in eradication plans for wild fauna [50].

Although there is currently one vaccine candidate that has already been approved in Vietnam [36], we believe that ASFV-989 is a promising vaccine, as it has not been obtained by genetic manipulation, which makes it easier to be applied through open distribution in the wild. Moreover, more than one gene was deleted, which might confer less risk of this strain reverting to virulence.

## 5. Patents

The results presented in this manuscript have been the subject of a European patent application, N° EP 4 036 226 A1, published 3 August 2022, bulletin 2022/31.

## Figures and Tables

**Table 1:** Experimental design for the in vivo studies.

Trial #	Group Number	Group Name	Nb of Pigs	Inoculation (D0)	Challenge	Necropsy
**1**	**A1**	**Control**	3	/	/	D100
	**B**	**989 ON LT**	5	989 ON	/	D100
	**C**	**989 IM LT**	5	989 IM	/	D100
	**D**	**989 IM/Geo IM D28**	6	989 IM	Georgia IM D28	D68
	**E**	**Geo IM D0**	3	Georgia IM		D6
	**F**	**Geo ON D0**	4	Georgia ON		D6
	**G**	**Geo IM D28**	3	/	Georgia IM D28	D34
**2**	**A2**	**Control**	5	/	/	D54
	**H**	**989 ON/Geo ON D28**	6	989 ON	Georgia ON D28	D68
	**I**	**989 ON/Geo IM D28**	6	989 ON	Georgia IM D28	D68
	**J**	**Geo ON D28**	4		Georgia ON D28	D34
**3**	**A3**	**Control**	3	/	/	D54
	**K**	**989 IM/Geo IM D14**	6	989 IM	Georgia IM D14	D54
	**L**	**989 ON/Geo ON D14**	6	989 ON	Georgia ON D14	D54

ON—oronasal; IM—intramuscular; LT—long term; Inoculum dose: 989 ON and Georgia ON: 10^4^ HAD_50_/pig; 989 IM and Georgia IM: 10^3^ HAD_50_/pig.

**Table 2:** Detection of ASFV-989 and Georgia viremia after IM Georgia strain challenge of immunized or nonimmunized pigs (D28 PI).

		Day Post-Challenge															
Group	Pig #	−1		3		5		7		11		13		18		25	
		**PCR 505**	PCR 989	**PCR 505**	PCR 989	**PCR 505**	PCR 989	**PCR 505**	PCR 989	**PCR 505**	PCR 989	**PCR 505**	PCR 989	**PCR 505**	PCR 989	**PCR 505**	PCR 989
**Geo IM D28 (group G)**	**370**	**ND**		**6.1**		**8.8**		†		†		†		†		†	
	**532**	**ND**		**5.4**		**8.9**		†		†		†		†		†	
	**7301**	**ND**		**6.5**		**9.2**		†		†		†		†		†	
	**mean**			**6.0**		**8.9**											
**989 IM/Geo IM (group D)**	**130**		5.7	**ND**	5.4	**ND**	5.3	**ND**	5.1	**ND**	5.0	**ND**	5.1	**ND**	4.8	**ND**	4.6
	**151**		4.7	**ND**	4.6	**ND**	4.4	**ND**	4.2	**ND**	3.8	**ND**	4.2	**ND**	3.5	**ND**	3.7
	**159**		4.6	**ND**	4.4	5.7	4.3	**ND**	4.0	**ND**	4.0	**ND**	4.2	**ND**	3.9	**ND**	3.9
	**7313**		5.3	**ND**	5.3	**ND**	5.2	**ND**	5.0	**ND**	4.8	**ND**	4.7	**ND**	4.4	**ND**	4.4
	**mean**		5.1		4.9		4.8		4.6		4.4		4.6		4.1		4.1
**989 ON/Geo IM (group I)**	**7357**		5.2	**ND**	4.6	**ND**	4.4	**ND**	4.1	**ND**	3.7	**ND**	3.6	**ND**	3.3	**ND**	3.3
	**7374**		5.2	**ND**	4.8	**ND**	4.7	**ND**	4.7	**ND**	4.0	**ND**	4.2	**ND**	3.1	**ND**	3.0
	**7386**		4.5	**ND**	3.8	**ND**	3.8	**ND**	3.2	**ND**	**ND**	**ND**	2.7	**ND**	**ND**	**ND**	**ND**
	**7391**		5.7	**ND**	5.0	**ND**	5.0	**ND**	5.2	**ND**	4.6	**ND**	5.0	**ND**	4.9	**ND**	4.2
	**7399**		5.2	**ND**	4.3	**ND**	4.5	**ND**	4.6	**ND**	4.0	**ND**	4.0	**ND**	4.0	**ND**	3.4
	**7401**		5.2	**ND**	5.0	**ND**	4.6	**ND**	4.6	**ND**	4.2	**ND**	4.5	**ND**	4.1	**ND**	3.9
	**mean**		5.2		4.6		4.5		4.4		4.1		4.0		3.9		3.6

ND—not detected; number—log10 eq HAD_50_/mL determined with PCR 505 and PCR 989; †—dead pig.

**Table 3:** Detection of ASFV-989 and Georgia viremia after ON Georgia strain challenge of immunized or nonimmunized pigs (D28 PI).

		Day Post-Challenge															
Group	Pig #	−1		3		5		7		11		13		18		25	
		**PCR 505**	PCR 989	**PCR 505**	PCR 989	**PCR 505**	PCR 989	**PCR 505**	PCR 989	**PCR 505**	PCR 989	**PCR 505**	PCR 989	**PCR 505**	PCR 989	**PCR 505**	PCR 989
**Geo ON D28 (group J)**	**7356**	**ND**		**4.4**		**8.8**		**†**		**†**		**†**		**†**		**†**	
	**7365**	**ND**		**3.3**		**8.4**		**†**		**†**		**†**		**†**		**†**	
	**7382**	**ND**		**4.2**		**9.3**		**†**		**†**		**†**		**†**		**†**	
	**7400**	**ND**		**4.9**		**9.4**		**†**		**†**		**†**		**†**		**†**	
	**mean**			**4.2**		**9.0**											
**989 ON/Geo ON (group H)**	**7358**		5.0	**ND**	4.5	**ND**	4.1	**ND**	3.9	**ND**	4.0	**ND**	3.2	**ND**	3.4	**ND**	2.6
	**7372**		5.2	**ND**	4.7	**ND**	4.4	**ND**	3.9	**ND**	3.7	**ND**	3.6	**ND**	2.4	**ND**	**ND**
	**7375**		4.9	**ND**	4.0	**ND**	4.1	**ND**	3.5	**ND**	3.6	**ND**	3.0	**ND**	**ND**	**ND**	3.4
	**7397**		5.2	**ND**	4.3	**ND**	4.3	**ND**	4.3	**ND**	4.2	**ND**	4.1	**ND**	3.9	**ND**	3.8
	**7403**		5.0	**ND**	4.0	**ND**	3.9	**ND**	3.9	**ND**	3.7	**ND**	3.4	**ND**	3.9	**ND**	3.3
	**7404**		4.7	**ND**	3.8	**ND**	3.4	**ND**	3.5	**ND**	**ND**	**ND**	3.2	**ND**	3.7	**ND**	**ND**
	**mean**		5.0		4.2		4.0		3.8		3.9		3.4		3.4		3.3

ND—not detected; number—log10 eq HAD_50_/mL determined with PCR 505 and PCR 989; †—dead pig.

**Table 4:** Detection of ASFV-989 and Georgia ASFV genome in tissues of immunized or nonimmunized pigs after challenge with Georgia strain.

Group	Pig #	Day Post-Challenge	Tonsil		Spleen		Hepato-Gastric LN	
			**PCR 505**	PCR 989	**PCR 505**	PCR 989	**PCR 505**	PCR 989
**989 IM/Geo IM (group D)**	130	41	**ND**	**ND**	**ND**	**ND**	**ND**	**ND**
	151	41	**ND**	**ND**	**ND**	**ND**	**ND**	41.0
	159	41	**ND**	36.8	**ND**	**ND**	**ND**	**ND**
	7313	41	**ND**	37.5	**ND**	39.7	**ND**	39.9
**989 ON/Geo IM (group I)**	7357	40	**ND**	ND	**ND**	40.9	**ND**	ND
	7374	40	**ND**	36.8	**ND**	ND	**ND**	ND
	7386	40	**ND**	38.1	**ND**	ND	**ND**	ND
	7391	40	**ND**	37.2	**ND**	ND	**ND**	43.6
	7399	40	**27.6**	ND	**36.7**	ND	**41.1**	ND
	7401	40	**32.6**	ND	**ND**	ND	**32.2**	ND
**989 ON/Geo ON (group H)**	7358	39	**ND**	**ND**	**ND**	**ND**	**ND**	**ND**
	7372	39	**ND**	30.9	**ND**	41.9	**ND**	**ND**
	7375	39	**ND**	**ND**	**ND**	39.6	**ND**	**ND**
	7397	39	**ND**	**ND**	**37.5**	**ND**	**ND**	**ND**
	7403	39	**ND**	**ND**	**ND**	39.6	**ND**	39.8
	7404	39	**ND**	**ND**	**43.4**	40.8	**ND**	**ND**

ND—not detected; number—Ct value for PCR 505 or PCR 989.

**Table 5:** Detection of ASFV-989 and Georgia viremia in immunized pigs challenged with Georgia strain 14 days after immunization.

		Day Post-Immunization (Georgia Challenge à D14)																								
Group	Pig #	0	3	5	7	11	13		17		19		21		25		27		34		41		46		54	
		PCR 989	PCR 989	PCR 989	PCR 989	PCR 989	**PCR 505**	PCR 989	**PCR 505**	PCR 989	**PCR 505**	PCR 989	**PCR 505**	PCR 989	**PCR 505**	PCR 989	**PCR 505**	PCR 989	**PCR 505**	PCR 989	**PCR 505**	PCR 989	**PCR 505**	PCR 989	**PCR 505**	PCR 989
**989 IM/Geo IM D14**	**7883**	0.0	5.7	6.1	6.3	6.4	**ND**	5.7	**ND**	5.6	**ND**	5.4	**ND**	5.7	**ND**	5.5	**ND**	5.0	**ND**	4.7	**ND**	4.2	**ND**	4.2	**ND**	3.8
	**7890**	0.0	5.3	5.9	5.9	5.9	**ND**	5.7	**ND**	5.3	**ND**	5.3	**ND**	5.4	**ND**	5.3	**ND**	4.8	**ND**	4.5	**ND**	3.6	**ND**	3.0	**ND**	0.0
	**7891**	0.0	6.2	6.4	6.6	6.8	†		†		†		†		†		†		†		†		†		†	
	**7895**	0.0	5.8	5.9	5.9	5.4	**ND**	5.5	**ND**	5.2	**ND**	5.2	**ND**	5.1	**ND**	5.1	**ND**	4.7	**ND**	4.6	**ND**	3.1	**ND**	3.1	**ND**	4.0
	**7901**	0.0	4.8	6.3	6.1	5.9	**ND**	5.7	**ND**	5.6	**ND**	5.5	**ND**	5.5	**ND**	5.2	**ND**	4.7	**ND**	4.0	**ND**	3.8	**ND**	3.3	**ND**	0.0
	**7908**	0.0	6.2	5.8	5.6	5.3	**ND**	5.4	**ND**	5.2	**ND**	5.3	**ND**	5.1	**ND**	4.2	**ND**	4.1	**ND**	3.8	**ND**	0.0	**ND**	0.0	**ND**	3.1
	**mean**	**0.0**	**5.7**	**6.1**	**6.1**	**6.0**		**5.6**		**5.4**		**5.3**		**5.4**		**5.1**		**4.6**		**4.3**		**3.0**		**2.7**		**2.2**
**989 ON/Geo ON D14**	**7882**	0.0	3.2	6.4	6.5	5.8	**ND**	5.8	**ND**	5.8	**ND**	5.5	**ND**	5.3	**ND**	5.0	**ND**	4.5	**ND**	4.1	**ND**	3.5	**ND**	0.0	**ND**	3.5
	**7888**	0.0	4.0	5.9	5.5	5.7	**ND**	5.5	**ND**	5.3	**ND**	4.7	**ND**	4.8	**ND**	4.4	**ND**	4.1	**ND**	0.0	**ND**	3.6	**ND**	3.3	**ND**	0.0
	**7889**	0.0	0.0	6.0	5.7	5.8	**ND**	5.8	**ND**	5.3	**ND**	5.2	**ND**	5.3	**ND**	4.7	**ND**	4.0	**ND**	3.5	**ND**	3.7	**ND**	0.0	**ND**	3.4
	**7893**	0.0	0.0	6.0	5.9	6.2	**ND**	5.9	**ND**	5.4	**ND**	5.2	**ND**	4.7	**ND**	4.5	**ND**	4.2	**ND**	3.6	**ND**	3.4	**ND**	3.8	**ND**	3.0
	**7904**	0.0	0.0	5.8	5.2	5.1	**ND**	5.0	**ND**	5.1	**ND**	4.7	**ND**	4.6	**ND**	4.6	**ND**	4.5	**ND**	4.1	**ND**	4.1	**ND**	3.5	**ND**	3.1
	**7907**	0.0	0.0	5.4	5.3	5.2	**ND**	5.1	**ND**	4.8	**ND**	4.7	**ND**	5.0	**ND**	5.0	**ND**	4.5	**ND**	4.4	**ND**	0.0	**ND**	3.6	**ND**	3.4
	**mean**	**0.0**	**1.2**	**6.0**	**5.7**	**5.6**		**5.5**		**5.3**		**5.0**		**5.0**		**4.7**		**4.3**		**3.3**		**3.1**		**2.4**		**2.7**

ND—not detected; number—log10 eq HAD_50_/mL determined with PCR 505 and PCR 989, †—dead pig.

**Table 6:** Detection of ASFV-989 and Georgia ASFV genome in tissues of immunized pigs challenged with Georgia strain 14 days after immunization.

Group	Pig #	Day Post-Challenge	Tonsil		Spleen		Hepato-Gastric LN	
			**PCR 505**	PCR 989	**PCR 505**	PCR 989	**PCR 505**	PCR 989
**989 IM/Geo IM D14**	**7883**	**53-55**	**ND**	27.6	**ND**	32.5	**ND**	36.3
	**7890**	**53-55**	**ND**	40.5	**ND**	38.3	**ND**	38.5
	**7891**	**12**	**ND**	30.2	**ND**	25.9	**ND**	29.1
	**7895**	**53-55**	**ND**	31.7	**ND**	36.1	**ND**	35.0
	**7901**	**53-55**	**ND**	34.9	**ND**	37.2	**ND**	40.1
	**7908**	**53-55**	**ND**	35.2	**ND**	40.7	**ND**	ND
**989 ON/Geo ON D14**	**7882**	**53-55**	**ND**	ND	**ND**	39.7	**ND**	ND
	**7888**	**53-55**	**ND**	40.7	**ND**	43.5	**ND**	35.1
	**7889**	**53-55**	**ND**	38.8	**ND**	39.8	**ND**	34.9
	**7893**	**53-55**	**ND**	ND	**ND**	35.5	**ND**	35.7
	**7904**	**53-55**	**ND**	41.1	**ND**	ND	**ND**	ND
	**7907**	**53-55**	**ND**	30.7	**ND**	40.0	**ND**	36.1

ND—not detected; number—Ct value for PCR 505 or PCR 989.

## Data Availability

Data from the study are available upon reasonable request to the corresponding author. All sequence data were uploaded to the NCBI under the study accession number PRJNA784367.
